# Celiac autoantibody positivity in relation to clinical characteristics in children with type 1 diabetes

**Published:** 2020-02-19

**Authors:** Khalid Siddiqui, Shaik Sarfaraz Nawaz, Nada Hareb Al Sumri, Dhekra Alnaqeb, Asim AlQurashi, Muhammad Mujammami

**Affiliations:** ^1^University Diabetes Center, King Abdulaziz University Hospital, King Saud University, Riyadh, Saudi Arabia; ^2^Strategic Center for Diabetes Research, College of Medicine, King Saud University, Riyadh, Saudi Arabia; ^3^Family Medicine, Department of non-communicable diseases, Ministry of Health, Sultanate of Oman; ^4^Endocrinology and Diabetes, Department of Medicine, King Khalid University Hospital, College of Medicine, King Saud University, Riyadh, Saudi Arabia

**Keywords:** celiac disease, type 1 diabetes, anti-tissue transglutaminase, anti-endomysial

## Abstract

**Background:**

Type 1 diabetes is an autoimmune disorder with a high risk of celiac disease (CD).

**Aim:**

This study aimed to determine the celiac autoantibody status and the clinical characteristics among children with type 1 diabetes and autoantibody positivity for CD compared to those without serological evidence of CD.

**Materials and Methods:**

In this cross-sectional study, 240 children with type 1 diabetes underwent serological screening CD. Blood glucose, glycated hemoglobin (HbA1c), hemoglobin, calcium, phosphorous, Vitamin D, alanine aminotransferase (ALT), alkaline phosphatase (ALP), aspartate aminotransferase (AST), and gamma-glutamyl transferase (GGT) were evaluated. The participants were screened for human anti-endomysial antibody and human anti-tissue transglutaminase antibody.

**Results:**

Of the 240 children with type 1 diabetes, 66 children were antibody positive for either anti-endomysial or anti-tissue transglutaminase or both autoantibodies for CD. There were 36 (54.5%) female and 30 (45.5%) male children with the mean age of 15.5±2.1 years. The mean duration of diabetes was 6.8±3.8 years. Only 35 (14.6%) children were found to have serological evidence of CD.

**Conclusion:**

CD is associated with type 1 diabetes. Serological screening for CD autoantibody should be performed routinely in children with type 1 diabetes. There is discrepancy in screening CD with antibodies, so a prospective follow-up of this cohort is needed for endoscopic evaluation and histopathological examination of intestinal biopsy to confirm CD in this population.

**Relevance for Patients:**

Anti-endomysial and anti-tissue transglutaminase autoantibodies should be included for screening CD among children with type 1 diabetes. Patients should undergo an endoscopy to confirm a diagnosis of CD.

## 1. Introduction

Celiac disease (CD) is a chronic gluten-induced enteropathy. It is a rare gastrointestinal condition that affects children of any race, ethnicity, sex, and age [1]. In genetically predisposed persons, CD is activated and triggered by gluten present in barley, wheat, and rye. Type 1 diabetes and CD are common autoimmune diseases with common genetic and immunological features [2]. These features include the presence of the HLA-DQ-2 and HLA-DQ-8 genes associated with disease development and the presence of autoantibodies against tissue transglutaminase 2 (anti-tTG2) and endomysium. The prevalence of CD in the general population varies from 0.2% to 5.6% in various regions of the world and the reason for this variance is not been explored yet. High prevalence of CD was reported in Algeria general population (5.6%) [1]. The prevalence of CD is very high in type 1 diabetes individuals and it varies from 3% to 12%. Increased prevalence of CD is also documented in other autoimmune diseases such as autoimmune thyroid disease (3%), autoimmune liver disease (13.5%), Down’s syndrome (5.5%), Turner syndrome (6.5%), William’s syndrome (9.5%), IgA deficiency (3%), IgA nephropathy (4%), and juvenile idiopathic arthritis (1.5%-2.5%) [1]. A previous study identified the seroprevalence of CD among 1167 healthy adolescents and it was reported to be around 2.2% in young general population [3]. CD has been reported among school-going children with a prevalence of 1.5% [4].

At present, the European Society for Pediatric Gastroenterology, Hepatology, and Nutrition, American Diabetes Association, and the American College of Gastroenterology clinical guidelines recommend the use of celiac-specific autoantibodies serology as markers to identify enteropathy in type 1 patients with CD [5-7]. A previous study in patients with Arab ethnicity suggests that out of 106 T1D children, 24.5% of children were positive for tissue transglutaminase 2 and/or endomysial antibodies [8]. Similar to the earlier study, the positivity of autoantibody in Indian type 1 diabetics was suggested to be 11.1% [9]. Although CD is well-described in the literature, there is an insufficiency of clinical characteristics and demographical information regarding the autoantibody status of children with type 1 diabetes. Therefore, the aim of this study was to determine the celiac autoantibody frequency for tissue transglutaminase 2 (anti-tTG2) and endomysial antibodies and their relationship with clinical characteristics in the children with type 1 diabetes.

## 2. Methods

This is a cross-sectional study conducted in the University Diabetes Center, King Abdul Aziz University Hospital, King Saud University, Riyadh. The sample included 240 children (age range 10-19 years) with type 1 diabetes recruited for this study between January 2016 and November 2018.

Type 1 diabetes was diagnosed according to the following criteria: Acute-onset ketosis or ketoacidosis; insulin therapy; and who were diagnosed earlier with T1D autoantibodies. Participants who were on immunosuppressive treatment were excluded from the study. The study was reviewed and approved by the Institutional Review Board of College of Medicine, King Saud University, Riyadh (Ref No. E-11-486). Informed consent was obtained from parents/guardians as the study participants were children.

### 2.1. Clinical and laboratory data

Demographic data were collected from patients’ files and information was extracted on the following symptoms: Abdominal pain, chronic diarrhea, vomiting, constipation, weight loss, chronic fatigue, bone pain, numbness/tingling in the extremities, and seizures. The participants were subsequently examined and information on weight, height, body mass index (BMI), and dental enamel defects were collected. Blood samples were obtained and centrifuged to collect serum on the day of diagnosis. Clinical and laboratory tests, including glycated hemoglobin (HbA1c), hemoglobin, calcium, phosphorous, Vitamin D, alanine aminotransferase (ALT), aspartate aminotransferase (AST), gamma-glutamyl transferase (GGT), and alkaline phosphatase values, were evaluated by clinical biochemistry analyzer, Randox Daytona (Randox Laboratories, Crumlin, UK). The participants were tested for IgA (Catalog No. KA2110, Abnova, Taiwan). The participants were screened for human anti-endomysial antibody (EMA-IgA, Catalog No. E0781h, Wuhan Elabscience Co., China) and human anti-tissue transglutaminase antibody (tTG-IgA, Catalog No. E1830h, Wuhan Elabscience Co.) using an ELISA kit.

### 2.2. Statistical analysis

The results were reported as mean and standard deviation or as numbers and percentage. We compared different groups with CD-associated autoantibodies using Student’s *t*-test for continuous data and Chi-square test for categorical data. *P*≤0.05 was considered statistically significant. All statistical analyses were carried out using SPSS 21 (IBM, Armonk, NY).

## 3. Results

Two hundred and forty children were included in the study. Mean age at diagnosis was 15.5 years; 106 (44.1%) were male and 134 (55.9%) children were female. The mean duration of diabetes was 7.4±4.2 years while the mean percentage of HbA1c at the time of diagnosis was 10.6±2.2.

Out of the 240 screened children with type 1 diabetes, 66 were seropositive for CD (either EMA, tTG-IgA, or both antibodies positivity). Their mean age at diagnosis was 15.5±2.1 years. There were 36 (54.5%) female and 30 (45.5%) male children and their mean duration of diabetes was 6.8±3.8 years. There was no significant difference in age, gender, and BMI between antibody-positive and antibody-negative participants (p > 0.05). Laboratory parameters such as HbA1c, hemoglobin, calcium, phosphorus, Vitamin D, ALT, AST, GGT, and alkaline phosphatase did not differ between the groups (Table 1).

**Table 1:** Clinical and serological data of type 1 diabetic children with celiac disease.

Variables	Total subjects (*n*=240)	Antibody negative (*n*=174)	Antibody positive (*n*=66)	*P*-value
Age (years)	15.5±2.5	15.6±2.6	15.5±2.1	0.871
Sex (M/F) (n)	(106/134)	(76/98)	(30/36)	0.805
Duration of diabetes (Y)	7.4±4.2	7.6±4.3	6.8±3.8	0.184
Height (cm)	158.0±22.3	157.9±25.3	158.0±10.8	0.984
Weight (kg)	55.2±13.7	55.2±14.0	55.2±12.9	0.981
BMI (kg/m^2^)	22.3±4.2	22.4±4.4	21.9±3.7	0.455
HbA1c (%)	10.6±2.2	10.6±2.3	10.7±2.0	0.751
Calcium (nmol/L)	2.3±0.09	2.31±0.09	2.31±0.08	0.575
Vitamin D (ng/mL)	33.1±16.6	33.6±16.0	31.8±18.3	0.513
Hemoglobin (g/L)	137.2±12.9	137.6±12.4	136.3±14.4	0.486
Phosphorus (nmol/L)	1.3±0.2	1.30±0.24	1.30±0.23	0.783
ALT (U/L)	18.6±23.8	18.1±23.6	19.9±24.5	0.609
AST (U/L)	22.0±27.44	20.9±19.8	25.0±41.4	0.309
ALP (U/L)	191.2±113.0	186.8±113.0	202.9±113.2	0.325
GGT (U/L)	16.8±13.0	17.0±14.9	16.8±12.3	0.918
IgA (ng/mL)	5.9±3.9	5.5±3.9	7.3±3.7	0.687

Data are expressed as mean±SD. Abbreviations: BMI: Body mass index, HbA1c: Hemoglobin A1c; AST: Aspartate aminotransferase, ALT: Alanine amino transferase, ALP: Alkaline phosphatase; GGT: Gamma-glutamyl transferase, IgA: Immunoglobulin A. P≤0.05 is statistically significant

**Figure 1:**
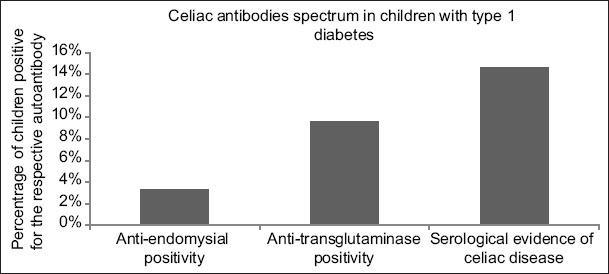
Serological data of type 1 diabetic children with celiac disease.

Sixty-six (27.5%) children were seropositive for either EMA, tTG-IgA, or both antibodies positive present at type 1 diabetes onset. The incident of multiple autoantibodies differed between the groups. Anti-endomysial positivity was a rare occurrence, appearing only in eight (3.3%) children. Conversely, anti-transglutaminase positivity was detected in 23 (9.6%) children. Only 35 (14.6%) children had serological evidence of CD and were found to be positive for both types of autoantibodies (Figure 1).

Table 2 demonstrates the clinical symptoms of type 1 diabetes children with CD. Sixty-six type 1 diabetes children were identified with CD. The clinical symptoms present in the celiac antibody group included diarrhea in 3 children (4.5%), chronic abdominal pain in 6 children (9.1%), fatigue in 20 children (30.3%), bone pain in 18 children (27.3%), seizure in 4 children (6.1%), dental discoloration and pitting in 10 children (15.2%), and numbness in 15 children (22.7%).

**Table 2:** Clinical symptoms of type 1 diabetic children with celiac disease.

Symptoms among CD patients	Antibody-negative group (*n*=174) (%)	Antibody-positive group (*n*=66) (%)	*P*-value
Diarrhea (yes)	8 (4.6)	3 (4.5)	0.986
Chronic abdominal pain (yes)	20 (11.5)	6 (9.1)	0.593
Fatigue (yes)	54 (31.0)	20 (30.3)	0.913
Bone pain (yes)	48 (27.6)	18 (27.3)	0.961
Seizure (yes)	8 (4.6)	4 (6.1)	0.642
Dental (brown discoloration, pitting (yes)	46 (26.4)	10 (15.2)	0.065
Numbness (yes)	46 (26.4)	15 (22.7)	0.556

Data are expressed as n (%). *P*≤0.05 is statistically significant

Sixty-six children who were seropositive for CD were divided into three groups: Anti-endomysial positive group, anti-transglutaminase positive group, and serological evidence of celiac disease group. There was no significant difference in age, height, weight, BMI, and diabetes duration among different groups when compare each group with without serological evidence of CD group. Laboratory parameters such as HbA1c, hemoglobin, calcium, phosphorus, Vitamin D, ALT, AST, GGT, and alkaline phosphatase also showed no significant changes in different groups (Table 3).

**Table 3:** Clinical and serological data of type 1 diabetes children with celiac disease classified into different groups.

Variables	Total subjects	Without serological evidence of CD	Anti-endomysialpositivity^b^	*P* value	Anti-transglutaminasepositivity^c^	*P* value	Serological evidence of CD^d^	*P* value
				
	*n*=240	*n*=174	*n*=8		*n*=23		*n*=35	
Age (years)	15.5±2.5	15.5±2.6	15.7±2.1	0.854	15.3±2.5	0.591	15.6±2.6	0.909
Sex (M/F)	(106/134)	(76/98)	(3/5)	0.730	(11/12)	0.707	(16/19)	0.825
Duration of diabetes (years)	7.4±4.2	7.6±4.3	8.2±4.0	0.698	6.9±3.1	0.468	6.4±4.1	0.129
Height (cm)	158.0±22.3	158.0±25.3	154.5±9.2	0.699	156.9±11.5	0.848	159.6±10.6	0.715
Weight (kg)	55.2±13.7	55.2±14.0	51.8±14.9	0.507	55.9±13.5	0.821	55.6±12.3	0.873
BMI (kg/m^2^)	22.3±4.2	22.4±4.4	21.4±4.4	0.507	22.5±3.9	0.953	21.7±3.5	0.402
HbA1c (%)	10.6±2.2	10.6±2.3	10.9±3.3	0.619	10.6±1.3	0.941	10.6±2.2	0.850
Calcium (nmol/L)	2.3±0.09	2.3±0.09	2.3±0.09	0.183	2.3±0.09	0.511	2.3±0.09	0.665
Vitamin D (ng/mL)	33.1±16.6	33.6±16.0	26.7±14.6	0.267	35.6±22.4	0.642	30.6±16.1	0.398
Hemoglobin (g/L)	137.2±12.9	137.6±12.4	130.8±13.6	0.137	137.4±9.2	0.941	136.7±17.3	0.743
Phosphorus (nmol/L)	1.3±0.2	1.3±0.2	1.4±0.3	0.206	1.4±0.2	0.253	1.2±0.2	0.221
ALT (U/L)	18.6±23.8	18.1±23.6	32.0±45.7	0.124	22.7±29.7	0.397	15.3±9.3	0.783
AST (U/L)	22.0±27.44	20.9±19.8	31.2±33.0	0.165	29.9±64.9	0.160	20.9±16.4	0.860
ALP (U/L)	191.2±113.0	186.8±113.0	203.5±118.2	0.683	206.4±108.5	0.432	200.4±118.2	0.517
GGT (U/L)	16.8±13.0	16.8±12.3	22.1±10.6	0.230	19.1±23.6	0.449	14.4±4.80	0.257

Data are expressed as mean±SD. Abbreviations: BMI: Body mass index, HbA1c: Hemoglobin A1c, AST: Aspartate aminotransferase, ALT: Alanine amino transferase, ALP: Alkaline phosphatase, GGT: Gamma-glutamyl transferase. For anti-endomysial positive group^b^, data were compared to without serological evidence of CD^a^. For anti-transglutaminase positive group^c^, data were compared to without serological evidence of CD^a^. For serological evidence of CD^d^, data were compared to without serological evidence of CD^a^. P≤0.05 is statistically significant

## 4. Discussion

The prevalence of Cd is quite high among Saudi children with type 1 diabetes (27.5%). We found that children with type 1 diabetes had higher occurrence of anti-TTG and anti-EMA antibodies (14.6%). On its own, anti-EMA antibody was rarely detected (3.3%), whereas some children were positive for anti-tTG antibodies (9.6%). The actual prevalence of CD is inappropriate to estimate because type 1 diabetes children may have atypical symptoms or none [10]. An earlier study reported that more than half of the Saudi pediatric population carries HLA-DQ genotypes that confer a higher risk of developing CD [11]. The prevalence of CD is high among patients with type 1 diabetes and may vary from 3 to 12% [1]. The prevalence rate reported in this study is also high and could be associated with genetic and environmental factors predisposing children to the development of CD. This study identified CD using two highly sensitive and specific antibodies, namely, anti-tTG and anti-EMA. Type 1 diabetic children may have false-positive anti-tTG low values, so the determination of lower positive cutoff value of anti-tTG may help in differentiating unusual variants [8]. In our study, 66 patients had autoantibody positivity and only 35 patients had serological evidence of both anti-tTG and anti-EMA positivity and had discrepant findings. There was a large discrepancy observed between the serological tests performed. To address this discrepancy, prospective follow-up of this cohort in children is needed for endoscopic evaluation and histopathological examination of intestinal biopsy to confirm CD.

Consequences of CD produces an improper T-cell-mediated immune response against ingested gluten in genetically predisposed children. Children with CD show an increased expression of HLA-DQ (*α*1*501, β1*02) heterodimer (HLA-DQ2) gene. When gluten is present, it triggers gliadin peptides present on the antigen-presenting cells to trigger intestinal mucosal T-cells. The tissue transglutaminase enzyme is one of the important targets of the autoimmune response in CD. The α-gliadin peptide neutral amino acid glutamine undergoes modification by host tissue transglutaminase to form negatively charged glutamic acids residues in α-gliadin peptide, which preferentially has a specific role in enhancing the α-gliadin T-cell response as well as dominant α-gliadin peptide T-cell epitope. These cause activation of lymphocytes to secrete pro-inflammatory cytokines such as interferon-γ, interleukin-4, and tumor necrosis factor-α, causing damage to the villi and resulting in enteritis. Moreover, elevated expression of HLA-DQ2 or DQ8 on cell surface antigens on the enterocytes may allow these cells to present additional antigens to the sensitized lymphocytes in the lamina propria [10].

The present study has some limitations. First, this was a cross-sectional study, thus we were unable to establish the causal relationship between CD and type 1 diabetes. Second, endoscopic and histopathological examination of intestinal biopsy to confirm the seropositive CD diagnosis was not performed. Third, serological measurement of antibodies was measured at a single time point.

## 5. Conclusion

CD is associated with type 1 diabetes. Serological screening for CD should be routinely performed among children with type 1 diabetes. There is a large discrepancy observed in screening for CD; an endoscopic evaluation and histopathological examination of intestinal biopsy is needed to confirm CD.

### Competing Interest

The author(s) declare that they have no competing interest.
